# Multi attribute method implementation using a High Resolution Mass Spectrometry platform: From sample preparation to batch analysis

**DOI:** 10.1371/journal.pone.0262711

**Published:** 2022-01-27

**Authors:** Sofia B. Carvalho, Ricardo A. Gomes, Anja Pfenninger, Martina Fischer, Michaela Strotbek, Inês A. Isidro, Nihal Tugçu, Patrícia Gomes-Alves

**Affiliations:** 1 iBET, Instituto de Biologia Experimental e Tecnologica, Oeiras, Portugal; 2 ITQB-NOVA, Instituto de Tecnologia Química e Biológica António Xavier, Universidade Nova de Lisboa, Oeiras, Portugal; 3 Sanofi R&D, Biologics Development, Industriepark Höchst, Frankfurt am Main, Germany; 4 Mammalian Platform, Global CMC Development, Sanofi, Framingham, MA, United States of America; Medimmune, UNITED STATES

## Abstract

Quality control of biopharmaceuticals such as monoclonal antibodies (mAbs) has been evolving and becoming more challenging as the requirements of the regulatory agencies increase due to the demanding complexity of products under evaluation. Mass Spectrometry (MS)-based methods such as the multi-attribute method (MAM) are being explored to achieve a deeper understanding of the attributes critical for the safety, efficacy, and quality of these products. MAM uses high mass accuracy/high-resolution MS data that enables the direct and simultaneous monitoring of relevant product quality attributes (PQAs, in particular, chemical modifications) in a single workflow, replacing several orthogonal methods, reducing time and costs associated with these assays. Here we describe a MAM implementation process using a QTOF high resolution platform. Method implementation was accomplished using NIST (National Institute for Standards and Technology) mAb reference material and an in-process mAb sample. PQAs as glycosylation profiles, methionine oxidation, tryptophan dioxidation, asparagine deamidation, pyro-Glu at N-terminal and glycation were monitored. Focusing on applications that require batch analysis and high-throughput, sample preparation and LC-MS parameters troubleshooting are discussed. This MAM workflow was successfully explored as reference analytical tool for comprehensive characterization of a downstream processing (DSP) polishing platform and for a comparability study following technology transfer between different laboratories.

## Introduction

Over the last decade, monoclonal antibodies (mAbs) have emerged as a major class of biological therapeutics, playing, nowadays, a key role in the pharmaceutical industry. Presenting a high rate of approval, the number of commercially available mAbs has been increasing drastically. As of 2019, more than 60 therapeutic mAbs have been marketing approved, which is a significant percentage of the global drug market. There are several mAbs in the top 10 of the best-selling drugs, generating annual revenues on the billion-dollar scale [1–3].

Most of the approved therapeutic mAbs are targeting oncological, immune-mediated, and infectious diseases. However, their applications are expanding into other indications [3–5]. New modalities are appearing and currently there are more than 80 mAbs in late-stage clinical studies [2]. The demand for new biotherapeutic products to treat unmet clinical needs together with mAbs’ potential is an indication that the mAb field will continue to grow. Therefore, it is expected that mAbs will persist as part of the therapeutic pipeline of the biopharmaceutical industry [5, 6].

In parallel with this growth, there is an increasing pressure to achieve high-quality products by improved development and manufacturing processes, which will not only meet high safety and efficacy requirements but also result in increased cost-effectiveness and high productivity. These aspects are important at all times but are particularly essential in the case of a pandemic, where there is a demand for extremely swift processes and rapid quality control [7–9]. Concerning these aspects, regulatory expectations have increased, and Quality by Design (QbD) strategies are now being used to guide the processes in the biopharmaceutical industry. This approach requires an in-depth product characterization and process understanding only possible through an accurate definition of critical quality attributes (CQA), “a physical, chemical, biological, or microbiological property or characteristic that should be within an appropriate limit, range, or distribution to ensure the desired product quality” [10] and critical process parameters (CPP), a variable that can impact the CQA. To address this need, companies are developing and implementing new and improved analytical tools, which are critical for enabling informed decisions during process development and manufacturing. These developments are subsequently contributing to improved design and control, decreased costs, and expedited bench-to-market timelines [6, 8, 9, 11, 12].

mAbs are structurally complex entities, quite different from small-molecule pharmaceuticals. These molecules are naturally highly heterogeneous and are subjected to several post-translational modifications (PTMs) during the manufacturing process. These product variants are strongly related to drug product safety and efficacy and must be characterized, from cell culture to long-term storage. Moreover, process parameters that can affect these PTMs must be identified and monitored in detail. Although it is critical to have in-depth knowledge of mAbs’ molecular attributes, their complexity is highly challenging in terms of analytical tools needed [1, 6–8].

A plethora of analytical methods are being used for characterizing protein modifications, i.e., product quality attributes (PQA) during mAbs’ discovery, development, and quality control stages. These tools combine immune assays, liquid chromatography, electrophoresis, biophysical, and mass spectrometry methods [6, 13–15]. Most of these methodologies, although widely used, are only able to evaluate one of the product’s characteristics, making full characterization costly and time-consuming [1]. Moreover, they are only able to monitor PQAs at the intact protein level and not at the amino acid level [8, 9]. CQAs may impact pharmacokinetics, binding properties, or immunogenicity, as they are product and application specific, their identification needs to be assessed by specific *in vitro* studies. Further *in vivo* studies are also required to confirm which attributes are indeed critical and should be included in the product specification criteria [16, 17].

Within this context peptide mapping mass spectrometry (MS)-based methods are widely used, as a powerful solution for primary structure characterization. The workflow of this bottom-up methodology consists of an enzymatic digestion, followed by peptide separation and MS detection. Within peptide mapping methods, multi-attribute method (MAM) approaches are becoming an emerging tool [6–8, 18]. MAM liquid chromatography (LC)-MS based method was firstly developed by Rogers et al [9] with the purpose to monitor simultaneously several critical attributes (chemical modifications) using only one method. Due to its promising potential, it has gained popularity and interest and several pharmaceutical companies have also developed and implemented MAM workflows [8, 18].

MAM can be divided into three practical steps: i) sample digestion into peptides; ii) sample analysis based on high resolution and accurate MS equipment; iii) data analysis using dedicated software for automated identification and relative quantification of targeted PTMs and detection of nontargeted new peaks [1, 9, 12]. This bottom-up approach has been used as an alternative to several time-consuming conventional assays, such as IEX, rCE-SDS, or HILIC glycosylation profiling. Charged-based modifications, protein fragments, glycosylation profiles, and other PTMs are monitored at the amino acid level, providing a more comprehensive analysis of product quality profile when compared to the individual orthogonal methods used in mAbs’ characterization. Although MAM is not able to provide information related to conformational changes (e.g., unfolding, partial reduction or protein fragments), that can be obtained with weak anion or strong cation exchangers analysis [19]. Importantly, MAM has also the ability to search for sequence variants, critical for mAb biological function [11, 20, 21]. Therefore, by streamlining monitoring and quality control analysis, MAM has the potential to accelerate mAbs’ process development and manufacturing, contributing to reduce the risk of failure and costs associated with analytical characterization during bioprocess development, product release, and in-process control [6, 9, 22]. MAM has been increasingly used for routine PQA analysis [18] keeping up with the growth and increased acceptance of MS methods in the biopharmaceutical field. In fact, according to a recent US Food and Drug Administration study, most of the biotherapeutics (with license applications approved between 2000 and 2015) use MS methods for their characterization. In line with this, recently, Song *et al* published the development and implementation of a MAM platform focused on global harmonization and intersite comparability [22].

However, to implement MAM at the quality control (QC) level, there are hurdles to overcome, some related to technical and/or regulatory issues or the capability of MAM to replace established release methods. In the technical space, sample preparation is a central aspect of MAM performance and reproducibility. To achieve reliable quantification of peptides with PTMs, it is critical to reduce the artificial modifications, mainly deamidations and oxidations, generated during sample preparation [1, 11, 12]. Moreover, the full integration of MAM workflows in the QC environment requires equipment, which is easy to maintain and operate. As QC personnel are not typically trained as mass spectrometrists, simple LC-MS methods, as well as user-friendly software and data readout, are also key factors for its success. This work describes the implementation and optimization process of a MAM platform using QTOF high-resolution equipment with SCIEX OS and BPV3.0 dedicated software for data analysis. Method implementation was performed using NIST (National Institute for Standards and Technology) mAb reference material (NIST-RM) and a real process sample (mAb S). PQA (chemical modifications) identification and analysis strategy, LC-MS parameters, and sample preparation optimization for batch analysis are also detailed. Application of the methodology for process samples, from distinct stages of the downstream process (DSP), was also explored targeting the PQAs evolution (removal/enrichment) during different purification stages.

## Results

### MAM implementation

#### NIST mAb reference material (NIST-RM)

A data-dependent LC-MS analysis (DDA) of the NIST-RM tryptic digest was performed using in-house previously established proteomics methods (see **Materials and Methods** **section**). The glycopeptide analysis allowed the identification of 15 glycan structures as well as the aglycosylated peptide (**Table 1**).

**Table 1:** NIST-RM glycan structures identified using MAM workflow and their relative abundance.

Oxford Notation	Monosaccharide Composition	Measured Relative abundance (%)		Literature values			
		High DP/CE	Low DP/CE	Lab1 [23]	Lab2 [23]	Lab3 [23]	TechNote [24]
FA2	H3N4F1	31.81	42.09	35.96	33.71	39.04	39.80
FA2G1	H4N4F1	32.19	37.98	30.04	39.22	33.00	36.83
FA2G2	H5N4F1	7.74	7.59	7.35	12.36	6.03	8.65
A1	H3N3	0.41	0.66	-	0.75	0.56	0.73
FA1	H3N3F1	13.32	3.46	14.96	3.40	10.40	3.33
FA1G1	H4N3F1	6.94	2.91	6.08	2.42	4.81	2.83
FA1G1Ga1	H5N3F1	1.43	1.33	0.98	0.96	1.25	0.99
FM5A1G1	H6N3F1	0.18	0.23	-	-	-	0.17
FA3G1	H4N5F1	0.59	0.45	-	1.09	0.97	0.44
FA3G2	H5N5F1	0.38	0.33	-	-	-	0.23
FA2G2Ga1	H6N4F1	1.16	1.30	1.01	3.16	1.29	1.50
FA2G2Ga2	H7N4F1	0.06	0.56	0.92	1.58	0.63	0.56
FA2G1Gc	H5N4Sa1	0.22	0.23	-	-	-	0.14
M5	H5N2	0.51	0.72	0.80	1.02	0.87	1.18
Aglycosylated	-	2.99	0.81	1.90	0.72	1.17	1.55

Glycan relative abundances measured using high Declustering Potential (DP)/Collision Energy (CE) and low DP/CE methods are listed. Monosaccharide Composition legend: H = Mannose/Galactose; N = N-Acetylglucosamine; F = Fucose; Sa = N-acetylneuraminic acid.

**Fig 1:**
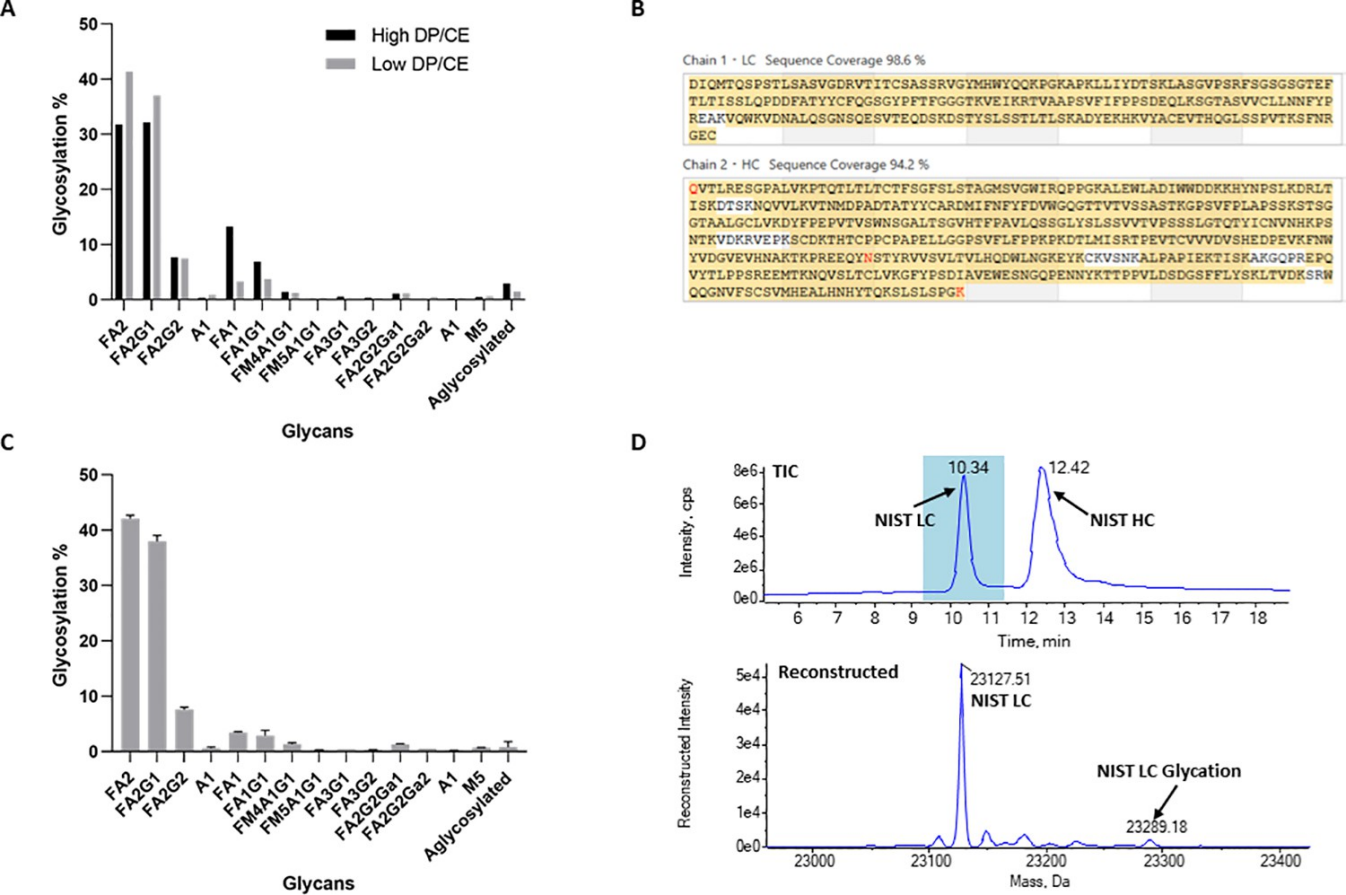
Implementation of the MAM workflow using the NIST mAb reference material. A) Comparison of the glycosylation profiles obtained using in-house previously established peptide ESI-ionization parameters (high DP/CE) and the optimized low DP/CE ionization method; B) Representation of the NIST-RM sequence coverage obtained with the low DP/CE ionization method considering the glycopeptide and MS/MS identifications; C) Repeatability of the glycosylation profiling using the low DP/CE ionization method. Error bars represent the standard deviation (n = 3); D) Light chain (LC) glycation monitored by intact protein analysis of reduced NIST-RM mAb. The peak corresponding to the glycated NIST-RM light chain (+ 162 Da) is indicated in the bottom panel.

First results (high DP/CE) suggested in-source fragmentation of glycopeptides. Comparing these results with the literature [24] four and two-fold increase in the percentage of the glycan structures FA1 and FA1G1 can be observed, respectively, concomitant with a decrease in the values of FA2 and FA2G1 glycans (**Table 1 and Fig 1A**). To overcome this issue, the ionization temperature was decreased from 500°C to 200°C, the Declustering Potential (DP) reduced from 80 V to 20 V, and the Collision Energy (CE) reduced from 10 V to 4 V. Glycosylation profiles obtained with high DP/CE and low DP/CE methods tested are represented in **Fig 1A**. A higher abundance for FA2 and FA2G1 glycans and a lower relative abundance of FA1 and FA1G1 glycans were observed when lower DP and CE were applied. For all the glycan structures detected, the obtained values using the low DP/CE method are aligned with the literature [23, 24] (**Table 1**). NIST-RM sequence coverage (glycopeptide + MS/MS) was 98.6% and 94.2% for the light and heavy chains, respectively (**Fig 1B**). We also evaluated the repeatability of the method by profiling the glycosylation pattern of three independent NIST-RM digests (**Fig 1C**). A standard deviation below 2% was observed for all measured glycopeptide relative abundances.

NIST-RM PQAs identification was done using BiopharmaView software and all were manually curated using MS and MSMS data and extracted ion chromatogram profiles (as described in **Material and Methods section**). The characterization results obtained were used to build a workflow for automatic detection and quantification of PQAs using the Analytics workspace in SCIEX-OS software. PQA relative abundance was calculated using the following formula:

$$Peptide\;modification\;(\%)=\frac{Peak\;area\;modified\;peptide\;x}{\sum peak\;areas\;of\;total\;peptide\;x}\times 100$$ (1)

**Table 2** lists the identified protein modifications and PQA relative abundances for the NIST-RM analysis. The obtained PQA relative abundances for NIST-RM are in agreement with published data [23]. Glycation is another PQA often monitored in mAb QC analysis. Subunit analysis with reduced NIST-RM was performed to analyse the relative levels of light chain glycation (**Fig 1D, top panel**). This protein modification affects the basic amino acids, mainly arginine and lysine. As a consequence, glycation induces missed cleavages when trypsin is used for protein digestion, hampering the relative quantification at the peptide level with the MAM sample preparation implemented. Therefore, light chain total glycation was detected in the reduced mAb by a specific mass increase of 162 Da (**Fig 1D, bottom panel**) and the peak area of the reconstructed protein species was used for its relative quantification. An average value of 3.68 ± 0.31% (average ± standard deviation, n = 3) was obtained for NIST-RM light chain glycation. Using peptide mapping data, glycation was detected at light chain K44, K52, K148, K182, K189 and K206. Site-specific glycation values assessed by peptide mapping using trypsin digestion were reported by Li et al and vary from ~0% to 3.8%. This highlights the limitations of using this enzyme when targeting glycation quantification, as discussed also by the authors [23].

**Table 2:** NIST-RM PQAs identified using MAM workflow and their relative quantification.

Modifications Light Chain						
Position	Amino acid	Modification	Measured Relative abundance (%)	Literature values [24]		
				Lab1	Lab2	Lab3
4	M	Oxidation	3.1	0.82	6.18	2.70
32	M	Oxidation	4.5	1.12	4.67	2.17
136/137	N	Deamidation	0.6	0.32	0.23	0.93
**Modifications Heavy Chain**						
1	Q	PyroGlu	>99.9	99.65	99.64	> 99
34	M	Oxidation	6.3	1.03	4.76	2.93
87	M	Oxidation	3.1	1.34	6.66	2.13
255	M	Oxidation	6.2	3.12	7.36	4.90
431	M	Oxidation	2.0	1.99	2.77	2.57
78	N	Deamidation	0.5	0.40	0.13	3.30
86	N	Deamidation	0.3	0.11	-	0.27
162	N	Deamidation	5.0	-	0.42	-
279/289	N	Deamidation	0.8	0.31	-	2.50
318	N	Deamidation	0.3	5.04	0.18	4.90
364	N	Deamidation	1.2	-	-	-
387/392/393	N	Deamidation	1.2	2.62	2.25 / 2.99	0.67
280	W	Dioxidation	1.5	-	0.58	-
316	W	Dioxidation	0.8	0.20	0.43	1.47
384	W	Dioxidation	0.4	-	0.75	-
450	K	Lys Loss@C-term	95.9	89.85	86.89	89.73

#### mAb S (IgG1 mAb, in-process sample)

To evaluate if the implemented MAM approach was directly applicable to in-process samples, mAb S PQAs were analysed using the same workflow. mAb S characterization, PQAs definition and automatic detection and quantification of those PQAs were performed as described for NIST-RM. High sequence coverage was obtained, with 98.6% and 92.7% for the light and heavy chains, respectively (glycopeptide + MS/MS). These values are similar to the values obtained for NIST-RM.

For this molecule the PQAs detected and monitored along the sample preparation optimization process were glycosylation profiles (**Table 3**), methionine oxidation, tryptophan dioxidation, asparagine deamidation, pyroglutamate (pyro-Glu) formation at the N-terminal and glycation **(Table 4)**.

**Table 3:** mAb S glycan structures identified using MAM workflow and their relative abundance (n = 9).

Oxford Notation	Monosaccharide Composition	Relative abundance (%)
FA2	H3N4F1	89.76 ± 0.22
FA2G1	H4N4F1	5.92 ± 0.14
FA2G2	H5N4F1	0.10 ± 0.01
FA1	H3N3F1	1.56 ± 0.06
FA3	H3N5F1	0.56 ± 0.04
A2	H3N4	0.47 ± 0.02
M5	H5N2	0.29 ± 0.03
Aglycosylated	-	0.52 ± 0.04

Glycan relative abundances are represented as average ± standard deviation. Monosaccharide composition legend: H = Mannose/Galactose; N = N-Acetylglucosamine; F = Fucose.

**Table 4:** mAb S PQAs identified using MAM workflow and their relative quantification (n = 9).

Modifications Light Chain			
PQA	Amino acid	Modification	Relative abundance (%)
Moxidation_1	M	Oxidation	0.51 ± 0.13
Ndeamidation_1	N	Deamidation	1.24 ± 0.62
Ndeamidation_2	N	Deamidation	0.33 ± 0.02
**Modifications Heavy Chain**			
PyroGlu_1	E	PyroGlu	0.39 ± 0.04
Moxidation_2	M	Oxidation	0.59 ± 0.06
Moxidation_3	M	Oxidation	2.09 ± 0.11
Moxidation_4	M	Oxidation	0^a^
Moxidation_5	M	Oxidation	0.71 ± 0.20
Ndeamidation_3	N	Deamidation	1.27 ± 0.04
Ndeamidation_4	N	Deamidation	0.79 ± 0.02
Ndeamidation_5	N	Deamidation	0.06 ± 0.00
Wdioxidation_1	W	Dioxidation	0.08 ± 0.04
Wdioxidation_2	W	Dioxidation	0.01 ± 0.00

PQA relative abundances are represented as average ± standard deviation.

^a^This PQA was detected only prior to the sample preparation optimization.

As for the NIST-RM, light chain glycation was monitored at subunit level (reduced mAb S) using intact protein mass analysis. We obtained an average value for total light chain glycation of 2.87 ± 0.09% (n = 4).

### Implementation of batch processing analysis

**Fig 2:**
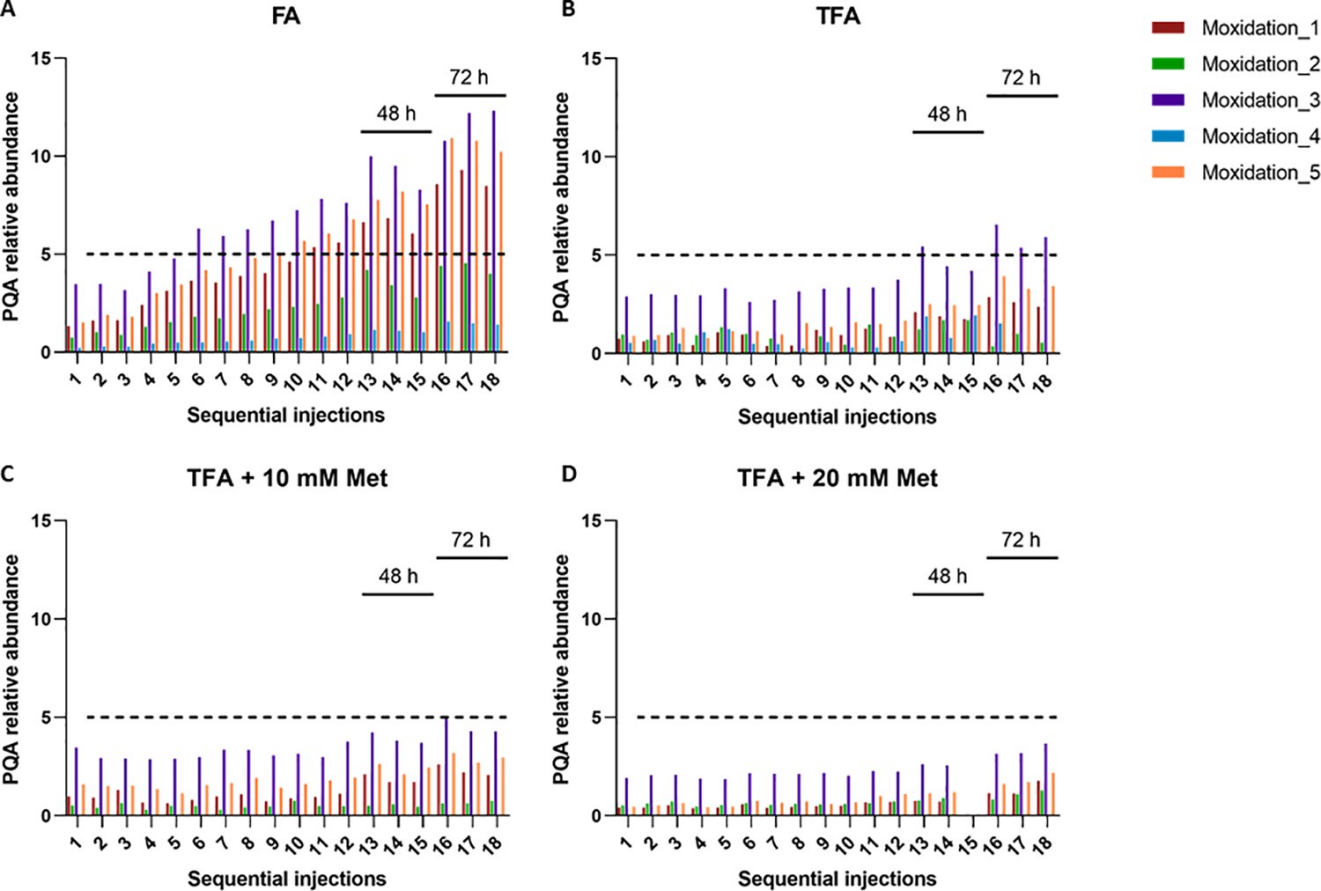
Implementation of the MAM workflow for batch processing analysis using Protein A purified mAb S. Sequential injections (until ~18 h, from injection 1 to 13) of the same sample were done to evaluate the stability of the oxidations at 4°C. After 48 h (injections 13, 14 and 15) and 72 h (injections 16, 17, 18) the same sample was re-analysed, as indicated in the different graphics of the figure. Different sample preparation protocols were tested: A) Formic acid (FA) was used to stop the trypsin digestion prior to LC-MS analysis; B) Trifluoracetic acid (TFA) was used instead of FA to stop trypsin digestion; C) Methionine at 10 mM was added during the denaturation step; D) Methionine at 20 mM was added during the denaturation step.

To be able to analyse multiple samples collected along the bioprocess, we further investigated the conditions that could guarantee PQAs stability in a batch processing mode. We first assessed sample stability during the time the sample was kept in the LC auto-sampler (at 4°C). Sequential injections of the same sample were done until a maximum of 72 h. As peptide oxidations are one of the PQAs more susceptible to artificial modifications generated during sample preparation [1, 13, 14], particular focus was given to the relative abundance of the five oxidation sites detected for mAb S (**Table 4**). We defined a threshold relative abundance of 5% as the maximum value acceptable for this PQA in this study. As can be observed in **Fig 2A**, oxidation levels start to increase even during the first 24 h and maintain this behaviour during the 72 h analysed. For Moxidation_3, the site where highest levels are observed, the oxidation relative abundance increases more than three-fold compared to the initial value and is well above the 5% threshold. A similar trend is observed for the other four oxidation sites, with Moxidation_1 and Moxidation_5 also achieving values higher than 5%.

In an effort to prevent artificially induced mAb oxidation and deamidation, modifications to the digestion protocol were made (**Table 5**). Formic acid (FA) was replaced by trifluoroacetic acid (TFA), at the same concentration, to stop the trypsin reaction and achieve a lower pH. The addition of TFA inhibits the increase in oxidation levels as compared to previous FA-treated samples (**Fig 2B and Table 5**). For one of the sites (Moxidation_4), the levels decrease to values close to zero. However, after 48 h in the auto-sampler (at 4°C), the oxidation levels of three sites start to increase and after 72 h, the Moxidation_3 site present oxidation relative abundances above the 5% threshold.

**Table 5:** Troubleshooting table summarizing issues found across MAM workflow implementation in this study, namely when focusing on sample batch processing. Possible reasons underlining the problems described and solution hints are also proposed.

Issue	Possible reason	Solution
**Glycoforms levels are higher/lower than expected**	In-source fragmentation of glycoforms. ESI-ionization temperature and voltage settings (declustering potential (DP) and collision energy (CE)) are too high	Decrease declustering potential, collision energy and ionization temperature
**Some PQAs are not unequivocally assigned (ex: oxidation, deamidation)**	Low abundant or not well assigned PQAs	Use stressed samples (example: alkylation step at high temperatures (70°C, 1h) and high pH >8 during digestion) to increase oxidation and deamidation levels in order to well characterize expected retention time and MSMS peak patterns
**High levels of oxidation/deamidation**	Artificial modifications due to sample manipulation during sample prep	Improve sample preparation protocol: decrease pH during digestion protocol (e.g. digestion buffer and to stop digestion); add methionine (evaluate concentration) at denaturation and after desalting steps; maintain samples at 4°C throughout sample preparation when not stated otherwise
**PQA levels are not stable during LC-MS batch run**	Sample stability	After sample preparation optimization referred above, maintain samples at 4°C in the LC autosampler. Divide samples analysis in different batches to avoid larger waiting times in the autosampler and freeze/thaw cycles. If needed, before LC-MS store sample digests at -80°C, however fresh samples are preferred
**Peak co-elution hampering the identification and relative quantification of deamination and isomerization**	Similar LC profiles and m/z signal	Optimize gradient/increase run length. Test different LC columns with lower particle size
**Glycation levels are too high when relative quantification is performed at the peptide levels using tryptic digests**	High missed cleavage rate	Perform glycation analysis at subunit level or at peptide level using a protease not specific to lysine residues

**Fig 3:**
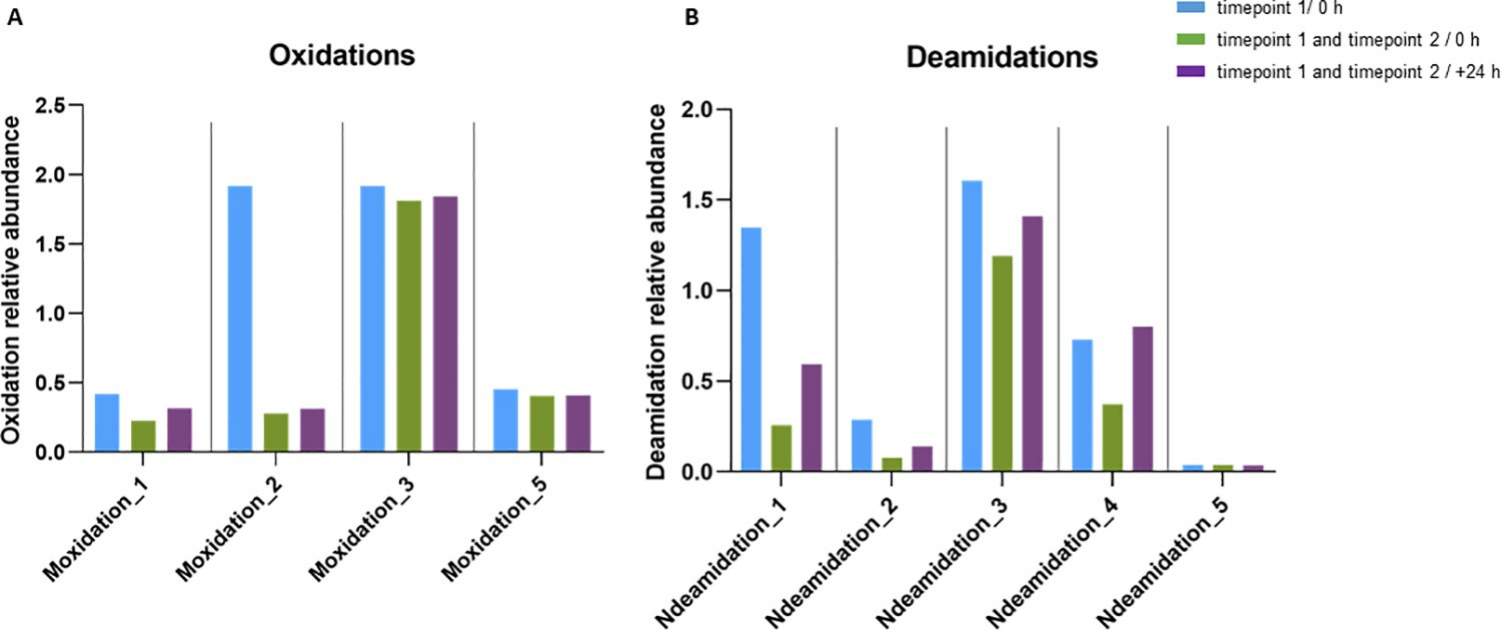
Impact of the addition of 20 mM methionine at two timepoints of the digestion protocol (timepoint 1: Addition at denaturation step and timepoint 2: After buffer exchange) on oxidation and deamination levels at specific sites of the molecule. Samples were analyzed immediately after the sample preparation procedure (0 h, green bars) and again after 24 h (purple bars). For reference, the levels of these PQAs obtained using the protocol with only 1 addition of 20 mM methionine (at the denaturation step, timepoint 1) are also plotted (blue bars).

Furthermore, methionine addition to the digestion protocol (at the denaturation step) was also evaluated (**Fig 2C and 2D**, **Table 5**), as the extrinsic methionine should act as an oxygen scavenger, maintaining the oxidation levels low and stable. Indeed, for digests treated with TFA and 10 mM methionine we observed oxidation levels below the threshold defined until reaching 48 h of analysis (**Fig 2C**). After 48 h, the levels tend to increase and are closer to the 5% relative abundance. The addition of 20 mM of methionine was also evaluated and retrieved better results, maintaining lower levels of oxidation for all five sites even after 72 h in the auto-sampler (**Fig 2D**). The stabilization of PQA levels was also observed for deamidation (**Fig 3**).

Since the digestion protocol involves a buffer exchange procedure (and therefore concomitant methionine depletion) we further evaluated a second addition of methionine immediately after this step. **Fig 3** compares the oxidation and deamidation levels after one addition of methionine (at the denaturation step, timepoint 1) analysed immediately after sample preparation (0 h) (**Fig 2D—**injection 1) and after two additions (at the denaturation step, timepoint 1, and after buffer exchange, timepoint 2), analysed immediately after sample preparation (0 h) and after 24 h. **Fig 3A** reveals that the double addition of methionine contributes to lower values already at the starting point that remain stable even after 24 h at 4°C. Similar results were obtained for N-deamidations but for the starting values, on the majority of the sites, the differences were even more striking when adding methionine twice (timepoint 1 and 2) (**Fig 3B**). However, this PQA presents lower stability when compared to oxidation, with the levels starting to increase at 24 h (**Fig 3B**). Importantly, all the conditions evaluated presented values that are far below the 5% threshold. Considering these optimization results, the final digestion protocol includes the use of TFA to stop trypsin digestion and the double addition of methionine at a 20 mM concentration.

MAM also enables detection of new peaks in a test sample versus a reference, potentially coming from process- and/or product-related impurities. In a new peak detection (NPD) method, we must distinguish between a new peak (that meets the defined minimal signal threshold and is unique to the test sample), a missing peak (that meets the defined minimal signal threshold and is unique to the reference sample) and a changed peak (detected both in the test and reference sample but with a difference in abundance that is above a user-defined fold change threshold) [25]. For the implementation of NPD, we used data from the oxidation stability study (4°C) with and without digestion protocol optimization (Fig 2A and 2D, respectively).

**Fig 4:**
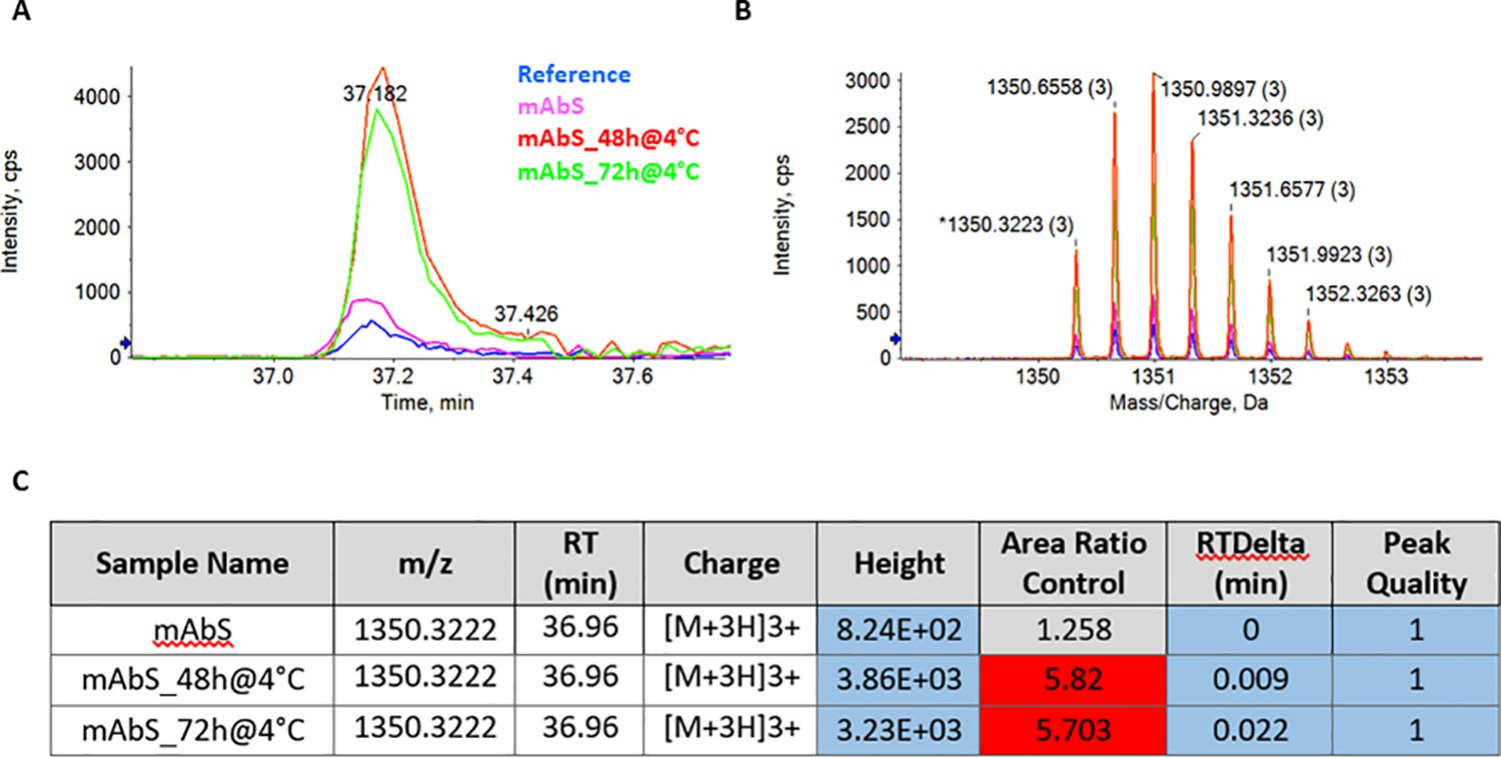
Implementation of New Peak Detection (NPD) workflow using the oxidation stability data (4°C). XICs data A) and mass spectra B) of m/z ion corresponding to Moxidation 5 of the reference, mAb S, mAb S_48h@4°C and mAb S_72h@4°C samples. C) MS data information showing the flagged peaks according to the defined criteria. Samples with a changed Moxidation_5 peak are highlighted in red (Fold changes ≥3). The NPD criteria highlighted in blue, indicate peaks that pass the criteria (peak height >500 counts, peak quality > 0.6, retention time delta ≤ 0.2 min). No flagged peak was detected for sample mAb S.

In our study the criteria used for NPD were: peak height >500 counts, peak quality > 0.6, retention time delta ≤ 0.2 min, +1 charge state peaks excluded and an area fold change ratio (test sample/reference) higher than 20. No components (peaks) meet the flag criteria of a new peak (or missing peak) in this analysis (**S1 Table**). To test further method applicability for the identification of changed peaks, we focused on the previously 5 identified oxidation sites and considered a fold change ≥ 3. In Fig 4, the NPD analysis for the Moxidation_5 is shown. Samples with fold change higher than 3 compared to the reference (were automatically flagged by the software (mAb S_48h@4°C and mAb S_72h@4°C). On the other hand, mAb S sample, which was analysed without any holding time at the autosampler (4°C), was not flagged according to the defined criteria, and so not considered as a changed peak when compared to the reference. Results for the other oxidation sites are shown in **S2 Table**. After the addition of TFA and 20 mM methionine to the sample prep protocol, no changed oxidation peak was detected (**S1 Table**).

### Threshold of detection, quantification and assay precision

A threshold for PQAs detection was defined using the following post-acquisition criteria: signal/noise ratio above 3 and a minimum signal intensity of 50 counts. According to this analysis, the detection threshold for our workflow was set at a relative abundance of 0.1%. Based on the MS data quality obtained in the implementation steps and considering the variability associated with the MAM method (sample preparation and LC-MS measurements) we empirically defined a threshold of quantification of 1%, below this we considered PQAs detectable (>0.1%) but not quantifiable.

**Fig 5:**
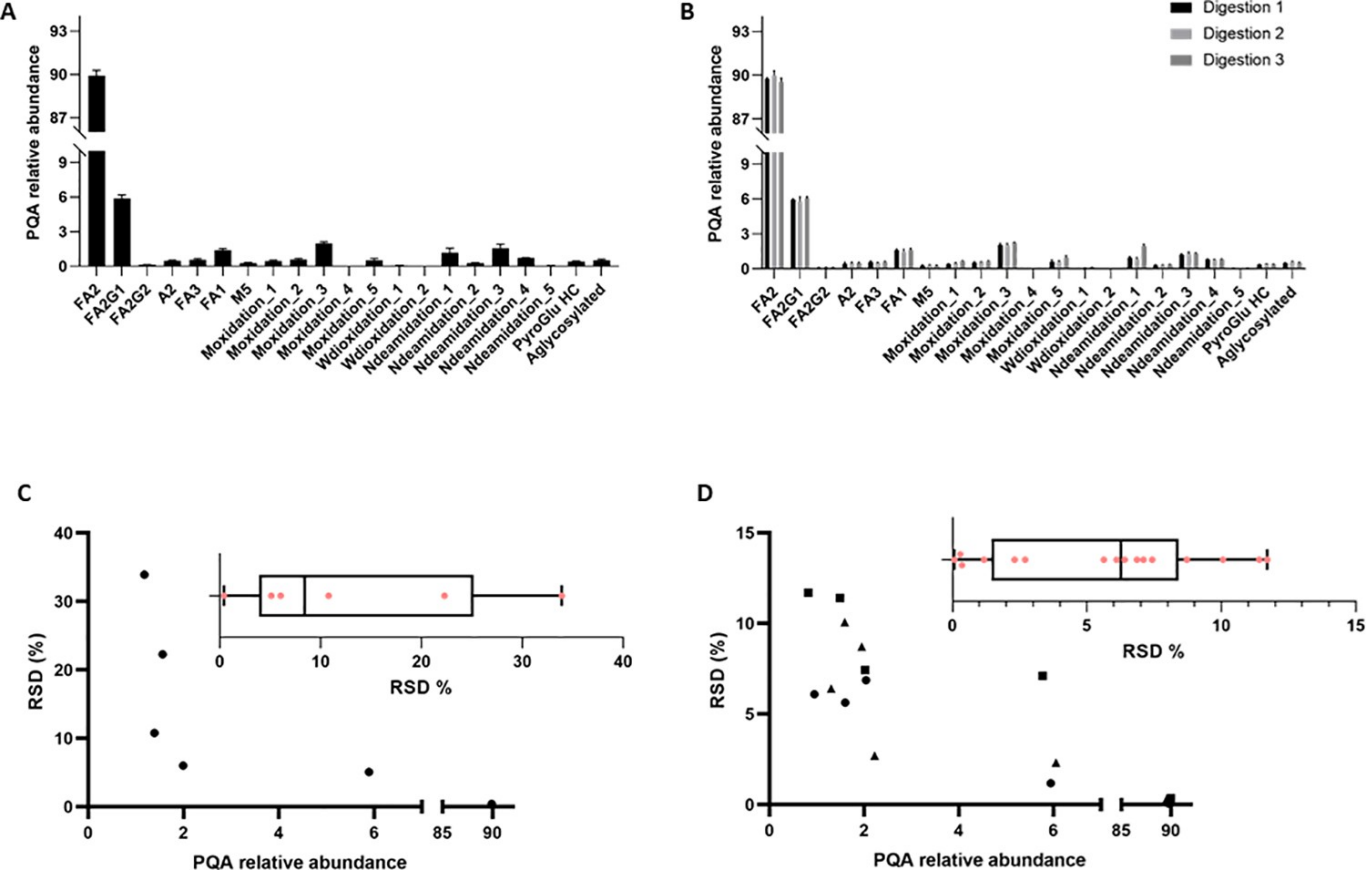
Determination of MAM assay precision using mAb S molecule. A) Inter-assay precision was evaluated by analyzing a single injection of 8 independent mAb S digestions (average PQA relative abundances are plotted). Error bars represent the standard deviation (n = 8). B) Intra-assay variability was determined by analyzing triplicate injections (average PQA relative abundances are plotted) from three independent trypsin digestions. Error bars represent the standard deviation (n = 3 for each of the digestions). C) Relative standard deviation (RSD) variation according to PQA relative abundance (above 1%) for inter-assay precision. RSD values for each PQA above 1% are presented in the box plot. D) RSD variation according to PQA relative abundance (above 1%) for intra-assay variability. Circle, square and triangle markers correspond to digestion 1, 2 and 3, respectively. RSD values for each PQA above 1% are presented in the box plot.

To determine the assay precision, we investigated the inter (assay-to-assay) and intra-assay (repeatability) variability (**Fig 5**). Inter-assay precision was evaluated by analysing 8 independent digestions, with one injection per digestion. Data were acquired with independent LC-MS sequences analysed on different days (**Fig 5A**). For the intra-assay, we performed triplicate injections from three independent trypsin digestions (**Fig 5B**). The raw data was analysed for PQA identification and relative quantification using the workflow previously established for mAb S. For PQAs that have relative abundances above 1%, the relative standard deviations (RSD) have an average value of 13.1% (ranging from 0.4 up to 33.9%) and 5.3% (ranging from 0.07 up to 11.4%) for the inter and intra-assay, respectively (**Fig 5C and 5D**). Deamidation_1 and Deamidation_3 are the only PQAs that present an inter-assay RSD higher than 20%, with values of 33.9% and 22.3%, respectively. As observed, RSD values are generally higher for PQAs presenting low relative abundance.

Precision data, thresholds of detection and quantification, and qualitative analysis of raw data enabled the definition of critical PQA variation ranges using as reference sample the Protein A purified mAb S. These variation ranges were further explored as pass/fail criteria for the high-throughput characterization of in-process samples (as discussed in the next section). Each threshold takes into consideration the signal intensity of modified peptides, translated into the PQAs relative abundances. For PQAs presenting relative abundances above 15%, we considered critical a variation of 1x standard deviation of the reference; for PQAs with relative abundances between 5 and 15%, we considered 1.5x standard deviation of the reference; for PQAs with relative abundances lower than 5%, we considered 2x standard deviation of the reference.

### In-process sample batch analysis: MAM applications

**Fig 6:**
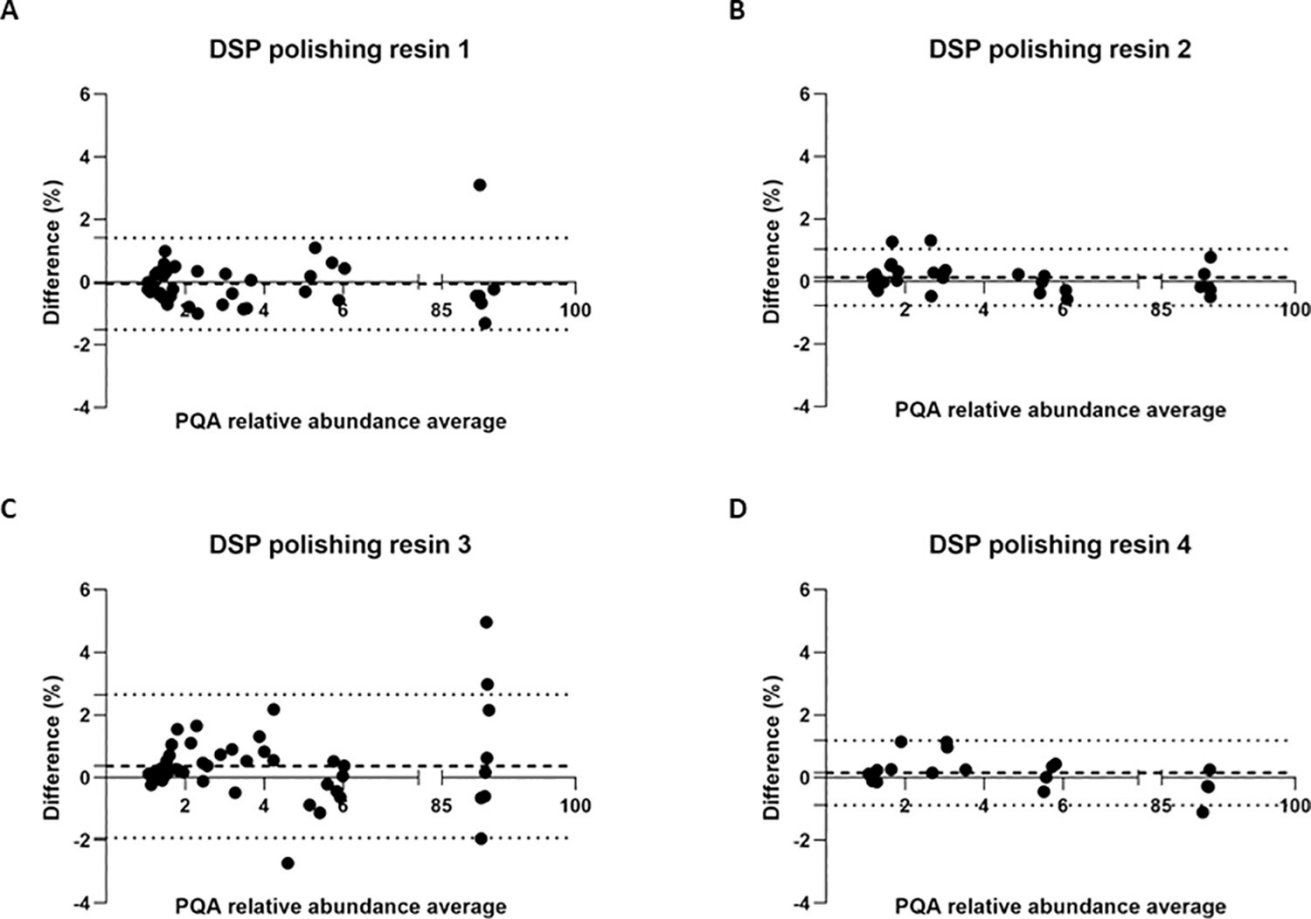
Bland-Altman plots to assess comparability of a polishing platform between two different laboratories. Differences between the PQA values (relative abundances >1%) obtained for 4 different polishing resins (A-D) at two purification scales (1 mL and 15 mL resin volume; n = 1 per scale and laboratory) are plotted (PQAs were evaluated in three selected fractions for each resin/scale). The dotted lines represent the limits of agreement given by the 95% confidence intervals for the mean bias, and the dashed line represents the mean bias from the Bland-Altman plot (the closest to zero the more similar are the results under evaluation, see section Statistical Analysis from Materials and Methods for more details).

First, we applied the established MAM workflow to do a comparability study of a polishing platform implemented at two different laboratories. Samples from distinct steps of the bioprocess were characterized. We used the pass/failed criteria described above to verify PQAs levels in 49 samples from 4 different resins (per laboratory). PQA profiles obtained indicated that samples from both laboratories presented similar values for the majority of the measured attributes as observed by the Bland-Altman plots presented in **Fig 6.** This analysis shows a very small mean bias (A: -0.05; B: 0.13; C: 0.37; D: 0.15) between both laboratories (the closest to zero the more similar are the two measures, see section **Statistical Analysis** from **Materials and Methods** for more details). We also observed in this analysis that most of the PQA values were within the limits of agreement (A: from -1.52 to 1.41; B: from -0.77 to 1.03; C: from -1.92 to 2.66; D: from -0.88 to 1.19). Very few exceptions were observed and those were very close to the critical range established by us (PQA profiles, pass/failed criteria) or defined by the statistical test (limits of agreement) (**Fig 6**).

**Fig 7:**
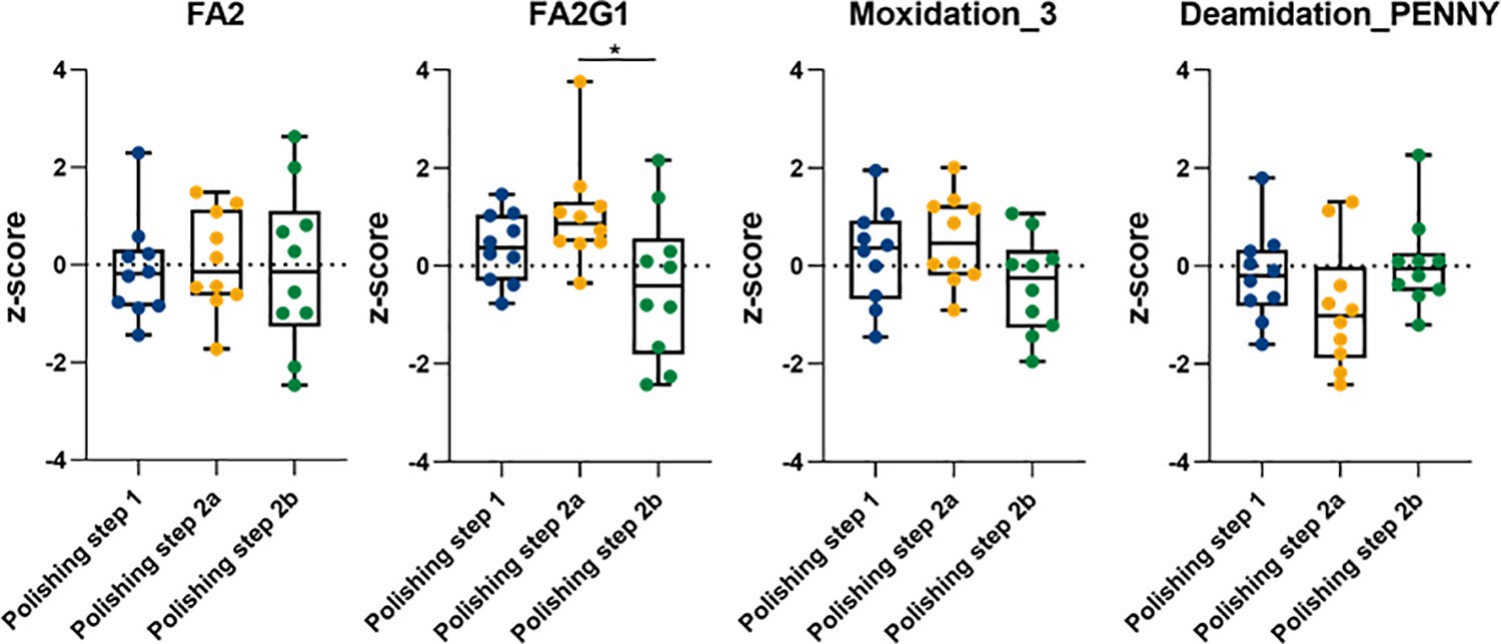
Level comparison for selected PQAs (FA2, FA2G1, Moxidation_3, Ndeamidation_3 or PENNY) monitored after purification using three different polishing resins, each one evaluated with 10 different buffer conditions. Polishing step 2a and 2b are two alternatives for the second polishing step. The values presented in the box plots are normalized according to the reference sample (z-score).

After guaranteeing the equivalence of the polishing platform, we evaluated how these DSP steps and different conditions (chromatographic and buffer conditions) impact product quality. We focused our analysis on 3 different polishing steps after protein A purification (polishing step 1 followed by 2a or 2b, representing two alternatives for polishing step 2). The main goal was to select conditions that maintain product quality with regards to PQA levels as compared to the reference (see the previous section). **Fig 7** shows the levels of 4 different PQAs, selected based on their intensity (relative abundances > 1%). FA2 and FA2G1 are the two main glycosylation structures present, Moxidation_3 and Deamidation at PENNY (Ndeamidation_3) peptide are the PQAs with higher values regarding the other PTMs analysed. Data were normalized according to the reference sample (z-score defined using the mean and standard deviation of PQA measurements, n = 2/polishing step, for the reference Protein A purified mAb S) for plotting. Additionally, as can be observed in **Fig 7**, each PQA do not present significant changes across different resins and 10 different process conditions. This is important to control product variations across the DSP. We also performed a Kruskal-Wallis non-parametric statistical test to evaluate if the medians of each PQA are different between DSP steps. The only significant difference was observed for the G1F glycosylation profile. A Dunns post-hoc test compared pairwise resins and identify that those significant differences are observed between polishing step 2a and 2b.

## Discussion

In this work, we implemented a MAM platform that provides mAb site-specific PTMs (e.g., profiling of glycan patterns, oxidations, deamidations, etc.) at the peptide level. This tool has been playing an important role within the biopharmaceutical field in the characterization of mAb PQAs (chemical modifications) and monitoring of possible CQAs (attributes whose levels may impact product quality), contributing not only to more efficient process development workflows, both for upstream and downstream, but also to attain better controlled bioprocesses and improved product quality control. One of the main challenges in realizing MAM full potential is to develop a robust, high-throughput method that can generate reproducible data in a batch processing mode. At method implementation one should consider not only the LC-MS equipment features, acquisition parameters, software and data analysis criteria, but also sample preparation robustness, sample stability and storage conditions.

Our workflow was implemented using a High-Resolution Mass Spectrometry platform (Sciex X500B QTOF system). This equipment was designed with a focus on biopharmaceuticals’ characterization, namely providing tools that enable a comprehensive mAb analysis. X500B workflows are simple, allowing high-throughput and streamlining routine analysis, which represent important factors to accelerate MAM integration into QC.

The product quality attributes of NIST-RM are extensively characterized and reported in the literature. The use of this reference material for implementation enabled a fine-tuning of the acquisition parameters by direct comparison to our results (**Tables 1, 2 and Fig 1A**) with published data [24]. The initial acquisition parameters were defined according to the in-house established proteomics protocols. However, these parameters impacted the quality of MAM data related to glycosylation profiles, as glycopeptides frequently suffer in-source fragmentation. This issue impacts the relative abundance of each glycan structure, by affecting the modified peptide quantification (Eq (1)), as fragmentation of one glycan structure can induce other detectable glycans. This glycan in-source fragmentation is a common event that occurs when using electrospray ionization MS equipment, and when this happens, ionization parameters need to be optimized [26]. In our study, redefinition of the ionization temperature, DP and CE parameters prevented the glycan in-source fragmentation we have previously observed, increasing the accuracy of the glycosylation relative levels (**Table 1**) and retrieving good repeatability results with a quite low standard deviation (below 2%) (**Fig 1C**). Accordingly, NIST-RM sequence coverage and all PQAs monitored in our workflow (**Fig 1**, **Tables 1 and 2**) are in agreement with the literature [24]. This technical issue and others are reported in **Table 5**, which guides the reader to possible solutions in a user-friendly, fast and readable way.

Glycation is also an important PTM to follow when working with mAbs, contributing to mAb heterogeneity (product charge), which may impact product stability and function (depending on the glycation site) [24, 27]. Regulatory authorities require its monitoring and quantification for product release. Although glycation can be measured using a MAM approach, here we used NIST-RM to implement a simple and fast intact mass analysis to quantify this PTM (**Fig 1D**), as also described by other groups [24]. The presence of glycation prevents trypsin cleavage at the C-terminus of the modified residue, resulting in the presence of glycated missed cleavage peptides and not enabling an accurate quantification of glycation. Our digestion procedure was optimized using trypsin, widely used in MAM approaches and optimal for the great majority of peptide modifications monitored [12, 13, 28–30]. It is, of course, possible to use other enzymes, but it requires a new workflow optimization for using an alternative.

MAM ability for high-throughput analysis is one of the main advantages for its use in DSP development and monitoring. Characterization of a mAb S reference sample was the starting point to define PQAs and establish the workflow for the analysis of in-process samples in batch processing mode (**Tables 3 and 4**). Batch analysis optimization, one of the main focus of this work, raised several issues that are reported in **Table 5**.

One of the critical points was sample stability while at the auto-sampler (4°C). This is highly relevant since we used LC gradients of approximately 60 min length, meaning that when a high number of samples need to be analysed, the last ones in the sample queue will stay at 4°C for a significant amount of time. Although we did follow all the PQAs reported in **Tables 3** and **4**, oxidation levels were the ones where this factor showed higher impact, with several values above the 5% threshold (**Fig 2A)**, probably because these PQA levels are reported as highly susceptible to artificial modifications [22, 31, 32]. Indeed, mAb S oxidation (and deamidation) levels are highly affected by the time the sample is waiting in the auto-sampler, presenting different fold changes depending on the amino acid where the modification is found (**Fig 2**). The sample digestion protocol was highly optimized to cope with these challenges. All the protocol modifications included, e.g., replacement of FA by TFA to stop enzymatic reaction (**Fig 2A and 2B**), addition of different concentrations of methionine and at different steps of the sample preparation procedure (**Figs 2C, 2D and 3**) contributed to achieving an improved sample preparation protocol, enabling a more robust and precise analysis. Artificial modifications such as oxidations and deamidations can be suppressed at low pH [27]. Replacement of FA (pKa 3.75) by a stronger acid such as TFA (pKa of 0.23) allowed to achieve an even lower pH by the end of the digestion procedure, which proved to be critical for the stability of the oxidation levels measured (**Fig 2B**). Moreover, extrinsic methionine added acts as an oxygen scavenger, maintaining the oxidation levels low and stable. The added concentration of this amino acid should be optimized according to the samples and the digestion protocol selected (**Fig 2C and 2D**). Although our digestion procedure proved to be robust, automated digestion implementation in a robotic workstation is an optimization to be considered, as it can improve throughput, accelerating batch analysis, and robustness, by eliminating operator errors during manual digestion [22]. Sample stability during sample digestion is key to take full advantage of robotic automated digestion.

Another relevant feature of MAM is the new peak detection (NPD) capability, which enables the application of MAM also as a purity assay during process monitoring, comparability assays or quality control applications. Although this was not our main focus, our final goal was to assess PQAs changing along a DSP polishing platform, we also defined a possible workflow for NPD. As expected in our dataset (data from sample stability at 4°C after digestion protocol optimization) no new peaks were detected, according to the NPD criteria defined. However, we were able to demonstrate the functionality of this workflow by automatically detecting changed peaks corresponding to oxidation modifications with an area fold change ratio (test sample/reference) higher than 3 (**Fig 4**), confirming the results discussed in **Fig 2**. NPD is mainly focused on detecting impurities and is independent of their identification. Noteworthy, in the case where new peaks are detected (m/z ion not present in the reference) MS data acquired as part of the NPD process can provide the necessary data to impurity identification. However, re-analysis of MS data must be performed for identification of those components.

MAM is a relative quantification method, which impairs an accurate definition of a limit of quantification (LOQ). In fact, to our knowledge, for this method, LOQ is not described in the literature or referenced by LC-MS equipment vendors. LOQs are product and PQA dependent, increasing the difficulties in establishing a true LOQ. Here, we defined an acceptable threshold for detection (0.1%) based on a signal-to-noise threshold ratio of 3 and a minimum signal intensity of 50 counts to assign a peak. As referred before, the definition of the quantification threshold (1%) was results-driven, based on LC-MS data quality and method variability (sample preparation and LC-MS measurements). Based on our experience and on the errors associated with other orthogonal methods we consider the precision RSD of both inter and intra-assays acceptable (< 20%) for PQAs above the threshold of quantification (**Fig 5**) and in line with what is reported in the literature for MAM [12]. The two PQAs that have values above 20% (Deamidation_1 33.9% and Deamidation_3 22.3%) are highly susceptible to artificial modification (sample handling, temperature, …), as discussed previously and reported in troubleshooting section (Table 5). and are very close to the 1% threshold, which can also contribute to the increased RSD values observed. These experiments highlighted the robustness of the developed MAM method, including the sample preparation protocol, the LC-MS analysis and the data analysis (**Fig 5A and 5B**). The implemented method has also a broad dynamic range (from 1 to 100%), allowing the monitor of different levels of PQAs in the same experiment.

In this study, we also explored the application of the MAM workflow to characterize mAb molecules’ PQAs and guarantee the comparability of a DSP polishing platform used in two laboratories. In a single MAM experiment, we were able to evaluate several PQAs, avoiding the use of a combination of different orthogonal methods (HILIC for glycan profiling, CEX for charged variants analysis, rCE-SDS for clipped variant analysis, for example) that would consume more time and resources (requiring for example, higher sample volume, different sample preparation procedures, equipment, columns and data analysis workflows, increased lab space, data storage capacity and instrument maintenance, supported by experienced human resources at the cost of losing sensitivity and specificity on the results). Our results showed very similar PQAs relative abundances between samples obtained at the two sites, confirming the comparability of both platforms (**Fig 6**). The mean bias for the measurements for all the DSP polishing resins are very close to zero, meaning that the differences in the values measured for both laboratories are very small. The limits of agreement, calculated by the Bland-Altman analysis, presented also small ranges, which reveals that the values of both measurements are close to each other. This type of study and MAM application can be critical to facilitate and accelerate technology transfer processes.

Moreover, we also used MAM to evaluate the DSP polishing platform transferred (polishing step 1 followed by 2a or 2b, representing two alternatives for polishing step 2) and characterize the effect of a range of chromatographic conditions (10 conditions evaluated per resin/step) on product quality. The impact of the different conditions tested was evaluated by monitoring the variation of PQAs levels when compared to the reference sample (Protein A purified mAb). We defined a critical pass/fail criterion for samples based on the PQA values of the reference and considering acceptable variation errors of 1x, 1.5x or 2x the standard deviation of the reference, depending on the intensity of the PQA (see **Results** section). As higher intensity PQAs (>15%) retrieve more accurate MS results, we defined a more restrictive error acceptance (1x standard deviation) for those. On the other hand, lower intensity PQAs are associated with higher variation (as they approach the thresholds of quantification and detection a higher probability of error is introduced) determining the tolerance of 1.5x or 2x the standard deviation, for PQAs within 5 to 15% and below 5% relative abundances, respectively. Samples from all steps of the polishing process and for different conditions were evaluated to check if the PQAs are within the expected range of relative abundances, which would mean that the different conditions/resins under study do not impact PQA profiles as reference levels are maintained stable across the polishing platform. The variation of these PQAs in the 10 conditions/resin tested was further evaluated on a Design of Experiments (DoE) to optimize and define the most suitable experimental conditions for the molecule under study. Moreover, PQAs monitored and quantified in our study (above 1% relative abundance) do not present major differences within each evaluated resin and across the 10 different process conditions per resin (**Fig 7**). The only significant difference observed was for the G1F glycosylation profile between polishing step 2a and 2b (we used Dunns post-hoc test to compare the resins pairwise). PQAs monitoring (by MAM and other complementary methods) throughout these polishing steps gave important hints on the selection of the best condition to purify mAb S and a more informed decision on the best alternative resin, key for efficient DSP development.

## Conclusions

Establishment of robust analytical tools that enable the characterization of PQAs and monitoring of possible CQAs is critical to achieve more efficient bioprocesses and safer products. The MAM platform implemented here proved to be suitable for in-process samples in a high throughput manner, which is of high importance during process development and monitoring.

The work developed here demonstrated the potential of MAM regarding several aspects: 1) it allows the replacement of several orthogonal methods, consuming less time and resources, while achieving an in-depth characterization of a broad range of PQAs in the same experiment; 2) it may include the detection of new peaks, an additional data processing function important to control product and process-related impurities, potentially enabling the detection of sequence variants or host-cell proteins; 3) it is proficient in PQA evaluation at different process stages (e.g., DSP) and conditions, even for wide dynamic ranges; 4) it enables the assessment of product comparability studies between different sites, which is key for efficient technology transfer.

We envisaged that all these powerful capabilities put MAM technology in a very good position to become one of the top analytical tools used in biopharma R&D and QC in a near future. As we come to better understand complex biopharmaceutical products and their CQAs, the design of therapeutic molecules and corresponding bioprocesses will itself become more efficient, excelling manufacturing pipelines and approval processes by regulatory authorities.

## Materials and methods

### Samples

MAM implementation was performed using two different antibodies, identified as NIST-RM and mAb S. NIST-RM is a mAb reference material (RM8671, National Institute of Standard and Technology), highly pure humanized IgG1κ expressed in murine suspension culture. mAb S is a molecule from an internal Sanofi project, a classical monoclonal antibody IgG1. This molecule was used in this study as a representative complex process sample, which was analysed at different purification stages.

### Chemicals and reagents

Digestion was performed using the following chemicals and reagents: Water LC/MS grade (Optima, Fisher Chemical W6), Trizma Base (Sigma, T1503), Trizma Hydrochloride (Sigma, T3253), Guanidine hydrochloride (GnHCl) solution (Sigma, G7294), DL-Dithiothreitol (DTT) (Sigma, 43815), Iodoacetamide (IAA) (Sigma, I1149), L-Methionine (Sigma, 64319), Sequencing Grade Modified Trypsin (Promega, PROMV5111), Trifluoroacetic acid LC/MS grade (TFA) (Optima®, Fisher Chemical, A116). Formic acid 0.1% solution in water LC/MS grade (Optima®, Fisher Chemical, LS118) and Formic acid 0.1% solution in acetonitrile LC/MS grade (Optima®, Fisher Chemical, LS120) were used for LC/MS runs.

### Sample preparation

Antibodies were subjected to trypsin digestion before peptide analysis. Briefly, samples were diluted in water to normalize concentration. 15 μg of monoclonal antibody was diluted in 6 M GnHCl, 25 mM Tris-HCl buffer pH 7.1 with 20 mM L-Methionine. Samples were reduced with 10 mM DTT for 15 minutes at 56°C, 400 rpm, followed by alkylation with 20 mM IAA for 30 min in the dark, at room temperature. The excess of IAA was quenched by adding 10 mM DTT, during 10 min in the dark at room temperature. A step of buffer exchange to 25 mM Tris pH 7.1 was performed using Zeba Spin Desalting Plates 7 kDa MWCO (Life Technologies, PIER89807), prepared according to manufacturer’s instructions. Samples were then digested with trypsin at a 1:50 W/W protein/trypsin ratio supplemented with 20 mM L-Methionine for 4h at 37 ˚C, 400 rpm. The digestion reaction was stopped by adding TFA to a final concentration of 1% before LC-MS analysis. Samples can be stored at—80 ˚C until further analysis.

The glycation profile was monitored at subunit level using reduced samples. For mAb reduction, samples were diluted in 6 M GnHCl, 25 mM Tris-HCl buffer pH 7.1 and incubated 15 min at 56°C with 10 mM DTT. Prior to MS analysis, samples were desalted with 10 kDa amicon filters using LC-MS Water with 0.1% FA.

### LC-MS analysis

All LC-MS experiments were performed in the X500B-QTOF (Sciex) equipped with the twin-spray ion source and coupled to the ExionLC AD UPLC system (Sciex). Tryptic peptides (5.6 μg injection on column) were separated using the bioZen™ 2.6 μm Peptide XB-C18 150 x 2.1 mm LC Column (Phenomenex). The following LC conditions were used: flow rate of 200 μL/min, column temperature 40°C, water with 0.1% formic acid as mobile phase A and acetronitrile with 0.1% formic acid as mobile phase B. The gradient was as followed: 1% B for 5 min; 1–10% B for 1 min; 10–35% B for 44 min; 35–60% B for 5 min; 60–90% B for 1 min; 90% B for 4 min: 90–1% B in 2 min; 1% B for 2 min. The column was washed by performing 2 cycles of 1–90% B for 10 min per cycle. Peptides were analysed by data dependent MSMS acquisition. The mass spectrometer was set for DDA full spectra scanning (300–1,800 m/z) for 300 ms. The top 10 ions were selected for subsequent MSMS scans (150–1,800 m/z for 100 ms each) using a total cycle time of 1.35 s. The selection criteria for precursor ions included dynamic background subtraction and counts above a minimum threshold of 100 counts per second. Ions were excluded from further MSMS analysis for 5 s. Fragmentation was performed using rolling collision energy with a collision energy spread of 5. ESI ionization parameters were: Ion source gas 1: 60 psi; Ion source gas 2: 60 psi; Curtain gas: 50 psi; Temperature: 200°C; Ion spray voltage: 5200 V; CAD gas: 7; Declustering potential: 20 V; Collision energy: 4 V; Time bins to sum: 4. MS was calibrated externally using the LC-MSMS analysis of a beta-galactosidase digest standard (Sciex), with 200 fmol injected on-column. The MS system was tuned prior to analysis using the ESI positive calibration solution for X500B (Sciex).

For the subunit analysis, reduced mAbs were separated using the Acquity UPLC Protein BEH C4, 300 A 1.7 μm 2.1x150 mm (Waters). The following LC conditions were used: flow rate of 200 μL/min, column temperature 60°C, water with 0.1% formic acid as mobile phase A and acetronitrile with 0.1% formic acid as mobile phase B. The gradient was as follows: 10% B for 3 min; 10–90% B for 10 min; 90% B for 2 min; 90–10% B for 1 min; 10% B for 5 min. X500B QTOF-MS was set to TOF-MS intact protein mode with TOF-MS m/z range of 600–3000, an ion accumulation time of 1 sec and the ion bins sum of 40. The ESI ionization parameters were: Ion source gas 1: 60 psi; Ion source gas 2: 40 psi; Curtain gas: 30 psi; Temperature: 500°C; Ion spray voltage: 5500 V; Declustering potential: 100 V; Collision energy: 15 V. The MS system was tuned as described above. A LC-MS system suitability test was performed using cythocrome C protein standard.

### Data analysis

MAM data were processed using BioPharmaView software 3.0 (Sciex) for product characterization and product quality attribute definition. BPV3.0 assay method was defined by specifying the protein sequence of interest and the digestion conditions (with a maximum of missed cleavages set to 1). The protein modifications considered were: deamidation of NQ; oxidation of M; dioxidation of MW; N-terminal Glu-pyroGlu of E and N-terminal Gln-pyroGlu of Q and C-terminal Lys-loss. The glycosylation profile was monitored considering the glycan structures presented in **Table 1**. Peptide assignments were done by correlation of MS and MSMS-level data, based on defined search parameters. For the peptide mapping settings, a peptide deconvolution tolerance of 10 ppm and a XIC m/z window of 0.025 Da was considered. For peptide assignments, it was considered a m/z tolerance of 5 ppm and a minimum MSMS score for auto-validation of 3, with a MSMS matching tolerance of 0.03 Da. After processing, peptide results were manually reviewed for NIST-RM and mAb S molecules. Peptide modifications automatically annotated were confirmed by data inspection and modifications not automatically annotated were assigned using pre-populated scoring results from processed data. Low abundance protein modification without MSMS confirmation were assigned after carefully manual analysis of the MS data, namely MS signal intensity and S/N ratio and also XIC profile. After the mAb characterization of a standard sample, the PQAs were defined and implemented for automatic batch processing. For PQA monitoring and quantification in batch processing, the Analytics workspace in SCIEX-OS software version 1.7 was used. The modified and non-modified peptides were defined and the PQAs relative abundance was defined as: Sum (peak area of modified peptide) / Sum (peak area of modified + non-modified peptide). The glycosylation relative abundance was defined as: Sum (peak area of glycoform) / Sum (peak area of all detected glycoforms). The integration parameters used in the Analytics workflow were: quantification and targeted identification using the MQ4 algorithm; minimum peak width of 3; Minimal peak height of 50; S/N integration threshold of 3; XIC width of 0.02; Gaussian smooth width of 1.0; Noise percentage of 40; baseline subtraction window of 2 and peak splitting of 2.

New peak detection analysis was implemented using the SCIEX-OS software 1.7 MAM-based workflow. The Analytics workspace was used with the non-targeting screening workflow to detect any component that is present in samples but not in control standards (quality control samples) or any component whose abundance is beyond a user defined range compared to control standards, thus serving as a purity check.

A new peak was defined using several criteria, namely peak height >500 counts, peak quality > 0.6, retention time delta ≤ 0.2 min and the fold change compared to a control sample. For the oxidation levels, a fold change > 3 as considered as a flag rule. For untargeted data processing, a fold change relative to a control sample > 20 was considered. In this untargeted analysis, singly charged ions were neglected. The software automatically flags all peaks that failed the user defined criteria.

The subunit MS data were analysed using the Intact protein analysis of BioPharmaView v3.0 software. The glycation was added as a user-defined modification affecting lysine residues with a mass increase of 162.0528 Da (molecular formula C_6_O_5_H_10_). The intact protein processing parameters were: matching tolerance ± 5 Da; m/z range from 400–3000 and a mass range defined by the theoretical protein species mass. The reconstruction processing settings were defined as: Iterations set to 20; S/N threshold ≥ 20; resolution set to 2500 and Gaussian smoothing of 1 point. Glycated light chain was identified by the 162 Da mass increase and the glycation relative abundance was calculated as: peak area of the reconstructed mAb light chain glycated / sum (peak area of the reconstructed mAb light chain protein species).

### Statistical analysis

Statistical analysis was done using GraphPad version 9.1.1. Comparability of the polishing platform between two different laboratories was assessed by using a Bland-Altman parametric analysis [33]. Having as input the PQA values measured in samples from both sites, the Bland-Altman method calculates the differences between both measurements (the mean bias), and 95% limits of agreement (confidence interval) for the mean difference (1.96x standard deviation). The limits of agreement can be used for visual judgement of how well both measurements agree. The smaller the range between these two limits the better the agreement is. Analysis of PQA variations across the DSP was performed using a Kruskal-Wallis non-parametric statistical test. A Dunns post-hoc test was used to identify the significant differences between individual pairs.

## Supporting information

S1 Table New Peak Detection (NPD) analysis for the Moxidations 1–5 after the addition of TFA and 20 mM methionine to the sample prep protocol, new peaks were considered when area fold change ratio ≥20. (XLSX)

S2 Table New Peak Detection (NPD) analysis for the Moxidations 1–5, changed peaks were considered when area fold change ratio ≥3. (XLSX)
